# Repetitive Exposure of IL-17 Into the Murine Air Pouch Favors the Recruitment of Inflammatory Monocytes and the Release of IL-16 and TREM-1 in the Inflammatory Fluids

**DOI:** 10.3389/fimmu.2018.02752

**Published:** 2018-11-30

**Authors:** Francesco Maione, Asif Jilani Iqbal, Federica Raucci, Michal Letek, Martina Bauer, Fulvio D'Acquisto

**Affiliations:** ^1^Department of Pharmacy, School of Medicine and Surgery, University of Naples Federico II, Naples, Italy; ^2^William Harvey Research Institute, Barts and The London School of Medicine and Dentistry, Queen Mary University of London, London, United Kingdom; ^3^Institute of Cardiovascular Sciences, College of Medical and Dental Sciences, University of Birmingham, Birmingham, United Kingdom; ^4^Health Science Research Centre, Department of Life Science, University of Roehampton, London, United Kingdom

**Keywords:** air pouch, chemokines, cytokines, IL-16, IL-17, inflammatory monocytes, TREM-1

## Abstract

The infiltration of Th17 cells in tissues and organs during the development of many autoimmune diseases is considered a key step toward the establishment of chronic inflammation. Indeed, the localized and prolonged release of IL-17 in specific tissues has been associated with an increased severity of the inflammatory response that remains sustained over time. The cellular and molecular mechanisms behind these effects are far from being clear. In this study we investigated the effects of two repetitive administration of recombinant IL-17 into the murine air pouch to simulate a scenario where IL-17 is released over time in a pre-inflamed tissue. Consistent with our previous observations, mice receiving a single dose of IL-17 showed a transitory influx of neutrophils into the air pouch that peaked at 24 h and declined at 48 h. Conversely, mice receiving a double dose of the cytokine—one at time 0 and the second after 24 h—showed a more dramatic inflammatory response with almost 2-fold increase in the number of infiltrated leukocytes and significant higher levels of TNF-α and IL-6 in the inflammatory fluids. Further analysis of the exacerbated inflammatory response of double-injected IL-17 mice showed a unique cellular and biochemical profile with inflammatory monocytes as the second main population emigrating to the pouch and IL-16 and TREM-1 as the most upregulated cytokines found in the inflammatory fluids. Most interestingly, mice receiving a double injection of IL-1β did not show any change in the cellular or biochemical inflammatory response compared to those receiving a single injection or just vehicle. Collectively these results shed some light on the function of IL-17 as pro-inflammatory cytokine and provide possible novel ways to target therapeutically the pathogenic effects of IL-17 in autoimmune conditions.

## Introduction

Interleukin (IL)-17 is the founding member of an evolving family of inflammatory cytokines whose functions remain poorly defined (1–3). Studies aimed at characterizing the physio/pathological functions of this cytokine both *in vitro* and *in vivo*, including our own (4–6), have shed some light on the unique role of this cytokine in inflammation (7). *In vitro*, IL-17 has been shown to induce the release of inflammatory mediators such as IL-6, IL-8, PGE_2_, MCP-1, and G-CSF by wide variety of cells, including fibroblasts (8), keratinocytes (9), epithelial (10), endothelial cells (11), and neutrophils (12, 13).

IL-17 *per se* is not considered a pathogenic cytokine but rather an amplifier of an inflammatory response. In fact, IL-17 has been reported to synergize with TNF-α for the induction of GM-CSF (14), or with CD40-ligand for the release of IL-6, IL-8, RANTES, and MCP-1 from a variety of cell types (15). All these findings are consistent with previous investigations where we and others have shown that IL-17 does not exert an inflammatory response when injected subcutaneously in normal non-inflamed tissue such as the soft pad of the paw of mice (6) and elicit only a mild inflammatory response when injected into the peritoneal cavity (6, 14). Conversely, injection of IL-17 in the pre-inflamed tissue of the air pouch caused a significant accumulation of neutrophils in the pouch and classical inflammatory cytokines such as IL-1β, IL-6, TNF-α, KC, and MCP-1 in the lavage fluid (6).

The fact that IL-17 causes the recruitment of PMNs to a tissue that is already inflamed, has been proposed to be one of the main mechanisms by which Th17 cells sustain and exacerbate inflammation e.g., via the recruitment of innate immune cells that would normally be present only in the initial phase of an inflammatory response (16–18). In this context, emerging evidences support the view that the infiltration and *in situ* differentiation of Th17 cells in inflamed tissues as the pivotal mechanism by which these cells cause damage to the target tissues (19–22). In line with this, seminal studies by Williams et al. proposed that the therapeutic effects of anti-TNF-α therapies in rheumatoid arthritis are linked to a “displacement” of Th17 cells. More specifically, treatment with anti-TNF-α caused a surprisingly increase in the number of Th17 cells circulating in blood—this being the result of a reduced infiltration of the Th17 cells in the inflamed joints with consequent improvement of the clinical score (23–26).

In this study, we sought to test the hypothesis that the presence of IL-17 at a local site of inflammation over a sustained period of time would initiate a unique series of cellular and biochemical events. To this aim, we tested the effects of repetitive administration of recombinant IL-17 into the pre-inflamed tissues of the air pouch model by injecting it at time 0 and once again after 24 h. Our results show that the repeated administration of this cytokine at a local site of inflammation prompts a unique inflammatory response featured by a 4-fold increase in the number of inflammatory leukocytes emigrated in to the pouch and a unique pattern of inflammatory mediators including CXCL13, IL-16, and TREM-1. Most importantly, none of these effects were observed with mice receiving double administration of IL-1β thus confirming our hypothesis about the unique biological role of IL-17 in inflammatory conditions.

## Materials and methods

### Reagents

Recombinant mouse IL-17A and recombinant IL-1β (from now on abbreviated as IL-17 and IL-1β) were purchased from R&D System (Abingdon, UK) and dissolved in carboxymethylcellulose (CMC 0.5% w/v). Unless otherwise specified, all the other reagents were from Sigma-Aldrich Co. (Dorset, UK).

### Mice

Male C57BL/6 mice (24–28 g; Harlan, UK) were used for all experiments. Animals were kept under standard conditions and maintained in a 12 h/12 h light/dark cycle at 22 ± 1°C in accordance with United Kingdom Home Office regulations (Guidance on the Operation of Animals, Scientific Procedures Act 1986), the European Union directives and following ARRIVE guidelines. All procedures were carried out to minimize the number of animals used and their suffering. All tests were conducted in a blinded fashion and according to the UK Animals Scientific Procedures Act, 1986. The local biological service unit at Queen Mary University of London approved all experimental protocols.

### Air pouch

Dorsal air pouches were prepared by injection of 2.5 ml of air on day 0 and day 3. On day 6, mice received the following treatments: 1- IL-17 (1.0 μg) in 0.25 ml of 0.5% CMC at time 0 and vehicle at 24 h (reported as IL-17 single injection); 2- IL-17 (1.0 μg) in 0.25 ml of 0.5% CMC at time 0 and at 24 h (reported as IL-17 double injection); 3- 0.25 ml of 0.5% CMC at time 0 and IL-17 (1.0 μg) in 0.25 ml of 0.5% CMC at time 24 (reported as IL-17 secondary injection); 4- 0.25 ml of 0.5% CMC at time 0 and at 24 h (reported as vehicle). The same experimental template was followed for IL-1β administration. In a separate set of experiments mice received a single IL-17 (2.0 μg) administration at time 0 or a single injection of the IL-17 (1.0 μg) 24 h post-vehicle injection at time 0. Mice were all sacrificed after 48 h from the first injection and air pouches washed thoroughly with 2 ml of PBS containing 50 U/ml heparin and 3 mM EDTA. Lavage fluids were centrifuged at 220 × g for 10 min at 4°C to separate the exudates from the inflammatory cells. Inflammatory exudates were collected and measured to evaluate the total volume and the level of inflammatory cytokines and chemokines, as described below. Total cell count was performed by light microscopy after staining in Turk's solution (crystal violet 0.01% in 3% acetic acid).

### Flow cytometry

Cells collected from the pouch cavities were first washed with PBS and then re-suspended in FACS buffer (PBS containing 1% FCS and 0.02% NaN_2_) containing CD16/CD32 FcγIIR blocking antibody (clone 93; eBioscience, Wembley, UK) for 30 min at 4°C. Thereafter, cells were labeled for 30 min at 4°C with the following conjugated antibodies (all from eBioscience, Wembley, UK): GR-1 (1:500; clone RB6/8C5), F4/80 (1:100; clone BMT), B220 (1:200; clone RA3-6B2), CD115 (1:200; clone AFS98), prior to analysis by FACS calibur using CellQuest software (Becton Dickinson, Franklin Lakes, NJ). At least 5 × 10^4^ cells were analyzed per sample, and determination of positive and negative populations was performed based on the staining attained with irrelevant IgG isotypes. Data were analyzed by FlowJo software.

### Cytokines and chemokines protein array

Equal volumes (1.5 ml) of the inflammatory fluids obtained were incubated with the Precoated Proteome Profiler array membranes (R&D Systems, Abingdon, UK) according to the manufacturer's instructions. Densitometric analysis of the dot plots was performed using the AIDA software from Raytest.

### Cytokine ELISA

Aliquots (100 μl) of the air pouch inflammatory fluids obtained at different time after IL-17, IL-1β or (their respective) vehicle injection were used neat or diluted 1:1 with assay diluents and analyzed for the levels of IL-1, IL-6, IL-16, IL-17, TNFα, and TREM-1 levels by ELISA according to the manufacturer's instructions (eBioscience, UK for IL-1, IL-6, and TNF-α and R&D system, UK for IL-16 and TREM-1).

### Statistical analysis

The results obtained were expressed as the mean ± SEM. Statistical analysis was performed by using one-way ANOVA followed by Dunnett's test when comparing more than two groups or two-way analysis of variance (ANOVA) for multiple comparisons followed by Bonferroni's test. In some cases, a student *T*-test was used to evaluate significance against the hypothetical zero value. Statistical analysis was performed by using GraphPad Prism 5.0 software (San Diego, CA, USA). Data were considered statistically significant when a value of *P* < 0.05 was achieved.

## Results

### Double injection of IL-17 into the air pouch exacerbates inflammation

**Figure 1:**
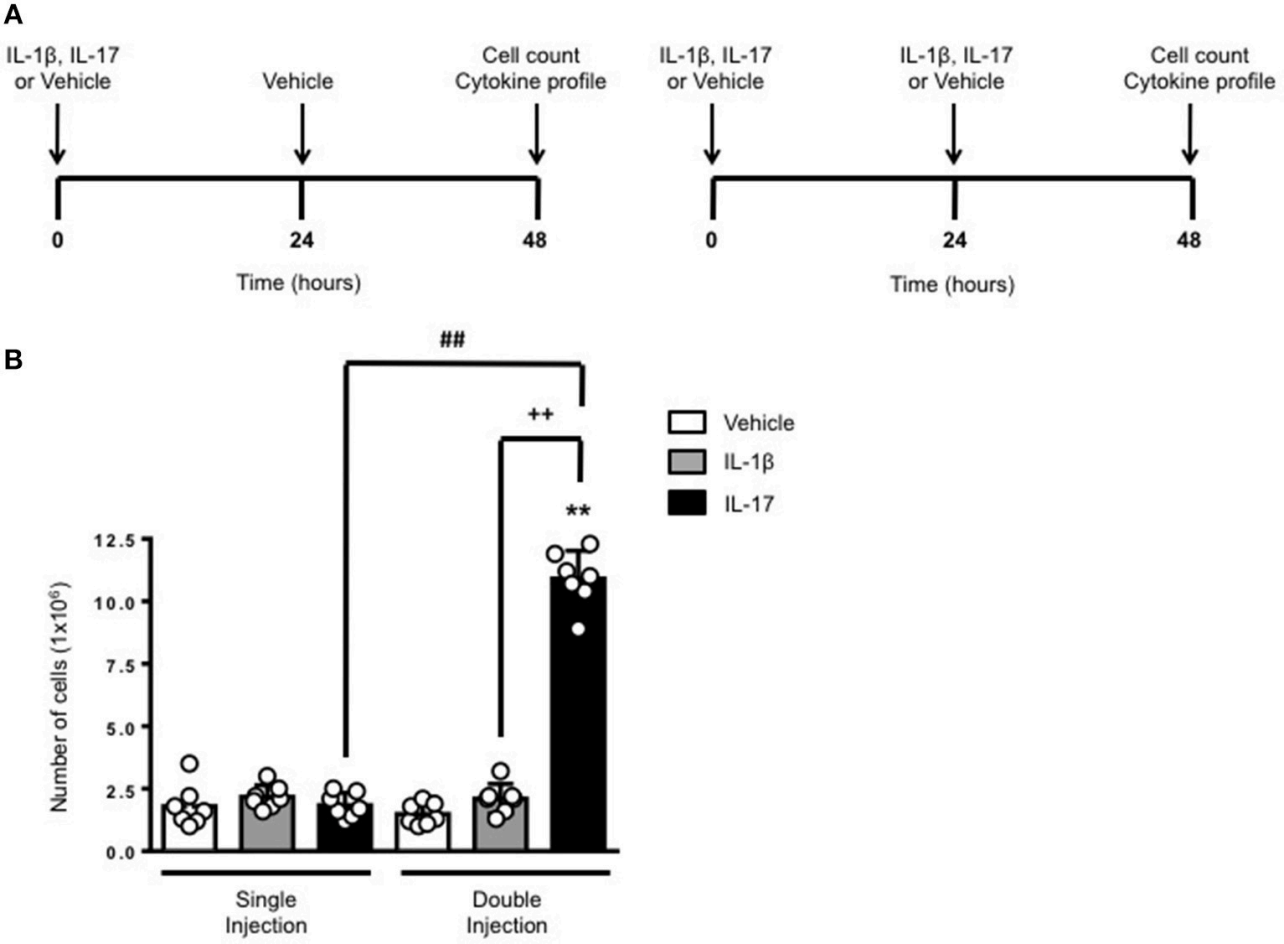
Effects of single and double injection of IL-17 in the air pouch. **(A)** Schematic representation of the experimental design used in this study. Male C57/Bl6 mice received two injections in the dorsal air pouch within 48 h. In the single injection protocol shown on the left, mice received a first injection of IL-1β or IL-17 (1.0 μg in 0.5 ml of 0.5% CMC) at time 0 and a second injection of vehicle (0.5 ml of 0.5% CMC) after 24 h. In the double injection protocol shown on the right, mice received a first injection of IL-1β or IL-17 (1.0 μg in 0.5 ml of 0.5% CMC) at time 0 and a second injection of the same inflamogen at the same concentration after 24 h. In both protocols mice were culled at 48 h after the first injection and then analyzed for the number of inflammatory cells migrated into the pouch. **(B)** Number of inflammatory cells recovered from the air pouch of mice that have received single or double injections of CMC vehicle (Vehicle; 0.5 ml) or the indicated inflamogens (1.0 μg in 0.5 ml of 0.5% CMC). Data are mean ± SEM, *n* = 7 animals per group of a single experiment and are representative of three independent experiments with similar results. Statistical analysis was conducted by two-way ANOVA with Bonferroni's multiple comparisons correction, **p < 0.01 vs. vehicle injection; ^##^*p* < 0.01 vs. IL-17 single injection; ^++^*p* < 0.01 vs. IL-1β double injection.

The schemes in Figure 1A show the experimental procedure we have used to compare a short (left panel) or long (right panel) exposure to inflammatory stimuli. We knew from previous studies (6) that a single administration of IL-17 into a 6-day old air pouch causes a transient infiltration of leukocytes that becomes evident at around 4–6 h, peaks at 24 h and then declines at 48 h. To test the effects of a continuous and localized release of IL-17 at the site of inflammation, we administered another dose of IL-17 or the same volume of vehicle at 24 h e.g., at the time the inflammatory response starts to fade away. Same experimental protocol was used for IL-1β or for vehicle-treated control mice. All mice were sacrificed 48 h after the first treatment.

Consistent with our previous findings, 48 h after a single injection of IL-17 or IL-1β mice showed no significant difference in the number of inflammatory leukocytes compared to vehicle-treated ones (Figure 1B). In contrast, mice receiving double injection of IL-17 but not IL-1β showed a marked increase (about 10 times more) in the number inflammatory infiltrates compared to either vehicle—or single injection of IL-17-treated mice thus providing first evidence for a sustained accumulation of inflammatory cells in tissues where IL-17 levels have been artificially kept at high levels. We also carried out differential counts to demonstrate that the main leukocyte population present at 48 h were monocytes in comparison to neutrophils and lymphocytes (Supplementary Figure 1). To ensure that the effects observed were a result of repeated exposure, and not the same as the acute response, we included mice injected with vehicle at time 0 and then IL-17 or IL-1β at 24 h prior to culling and analyzing at 48 h (Supplementary Figure 2). We found that there was a significant recruitment of leukocytes at 48 h following a single injection of IL-17 at 24 h, however, the level of recruitment was still significantly exacerbated in mice which had a received double injection of IL-17 when compared (Supplementary Figure 2).

In order to clarify whether the differing leukocyte recruitment profiles observed were dependent upon IL-17 concentration or repeated IL-17 exposure we administered a single injection of 2.0 μg IL-17 at time 0 in contrast to the established protocol of 1.0 μg at 0 and 24 h (Supplementary Figure 3). We found that the critical parameter was indeed repeated exposure rather than IL-17 concentration (Supplementary Figure 3).

**Figure 2:**
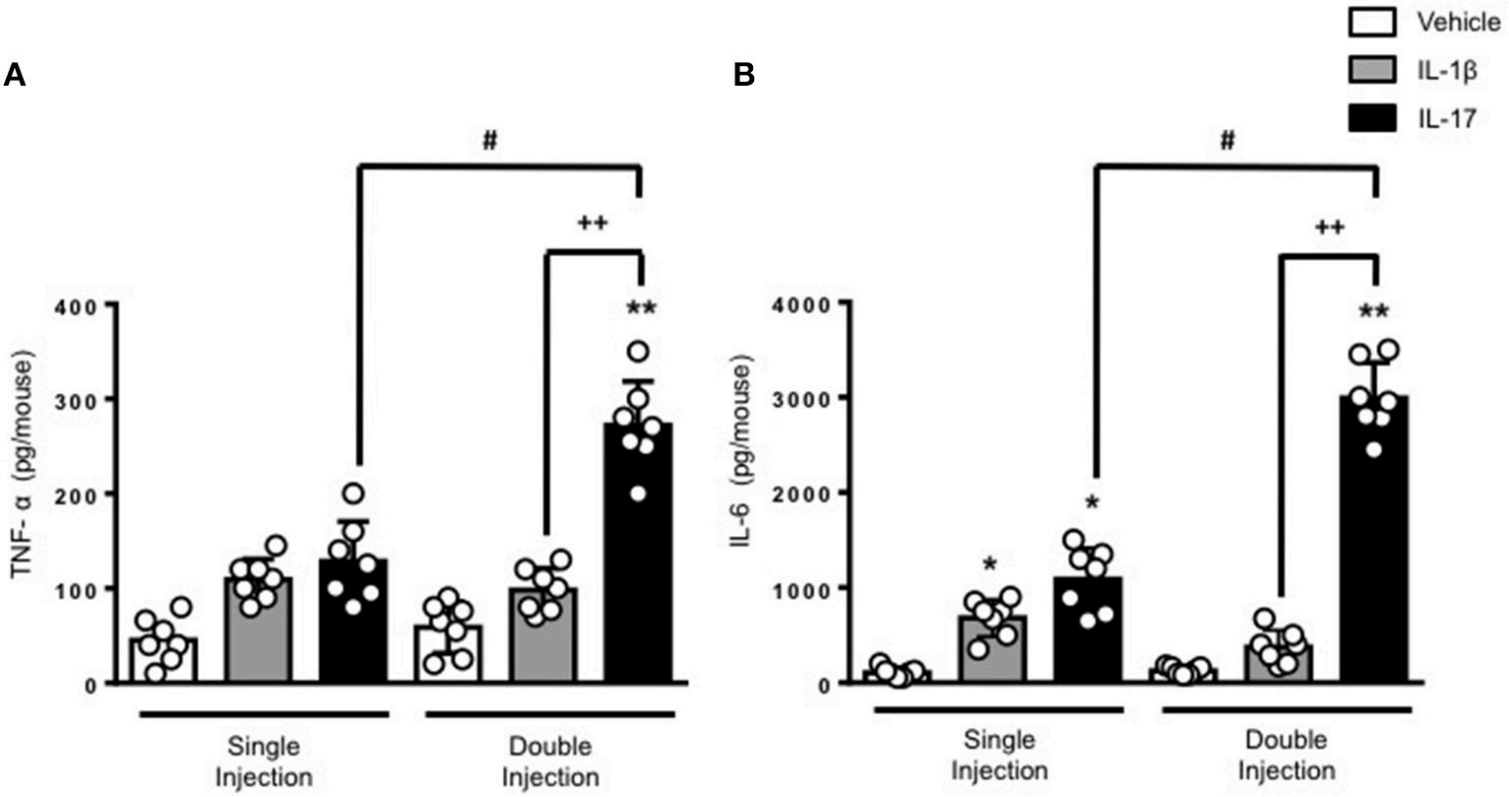
Levels of **(A)** TNF-α and **(B)** IL-6 present in the inflammatory fluids of mice receiving single or double injection of IL-1β or IL-17 into the air pouch. Male C57/Bl6 mice received a single or double injection of IL-1β or IL-17 (1.0 μg in 0.5 ml of 0.5% CMC) as described in Materials and Methods. The inflammatory fluids collected from the air pouch at 48 h were used to measure the levels of TNF-α and IL-6. Data are mean ± SEM, n = 7 animals per group of a single experiment and are representative of three independent experiments with similar results. Statistical analysis was conducted by two-way ANOVA with Bonferroni's multiple comparisons correction, *p < 0.05 vs. vehicle injection, **p < 0.01 vs. vehicle injection; ^#^*p* < 0.05 vs. IL-17 single injection; ^++^*p* < 0.01 vs. IL-1β double injection.

The results obtained on leukocyte recruitment were reflected in the levels of inflammatory cytokines in the air pouch fluids. Both TNF-α and IL-6 were significantly higher in mice receiving a double dose of IL-17 compared to mice treated with a single dose of IL-17 or vehicle (Figures 2A,B, respectively). Once again, we did not observe any significant difference in the levels of IL-6 and TNF-α between vehicle and double IL-1β–injected mice. We also confirmed that IL-17 was still present in the air pouch at 48 h after a single injection (Supplementary Figure 4).

### Double injection of IL-17 into the air pouch induces the recruitment of inflammatory monocytes

**Figure 3:**
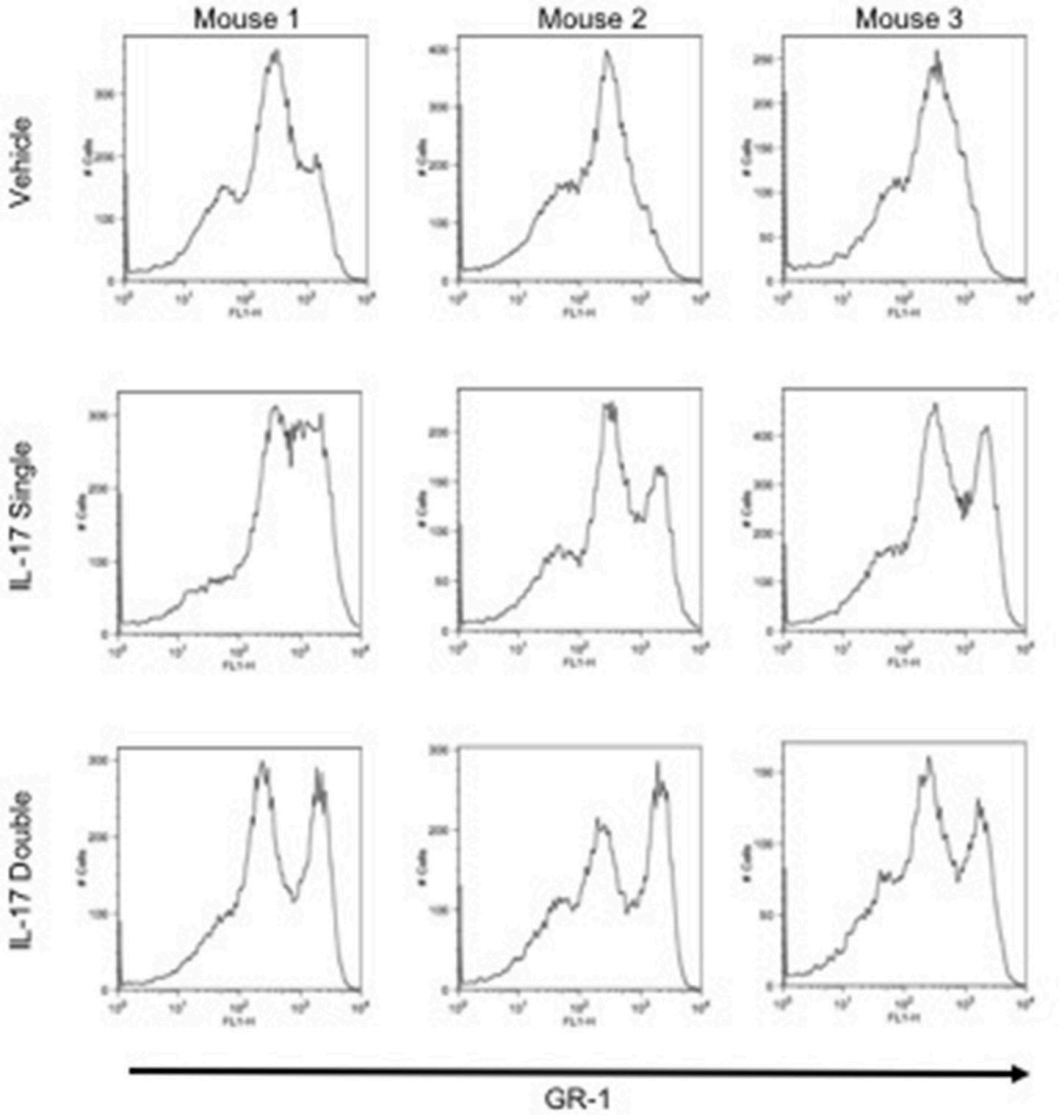
GR-1 sub-populations amongst the inflammatory cells collected from mice receiving single or double injection of IL-17 in the air pouch. Male C57/Bl6 mice received single or double injection of IL-1β or IL-17 (1.0 μg in 0.5 ml of 0.5% CMC) as described in section Materials and Methods. The inflammatory cells collected from the air pouch at 48 h were washed, stained with GR-1 and analyzed by FACS. At difference from mice receiving vehicle control that show one main population of GR-1+ cells, mice receiving single or double injection of IL-17 show the presence of two subpopulations: GR-1^high^ and GR-1^Int^. Results are from 3 independent mice chosen randomly from a single experiment and are representative of three independent experiments with *n* = 7 mice/treatment.

To investigate and compare the phenotype of the inflammatory leukocytes recruited by single or double injection of IL-17 we stained cells with an anti-GR-1, a pan granulocyte marker and analyzed them by flow cytometry. Interestingly, while vehicle-injected mice showed the presence of a single GR-1^int^ population, mice receiving a single or double injection of IL-17 had two populations: a GR-1^int^ and GR-1^high^ (Figure 3, middle and bottom panels, respectively). Given that GR-1 expression levels have been previously used to distinguish neutrophils (GR-1^high^) from resident (GR-1^+^) or inflammatory (GR-1^−^) monocytes (27–29), we adopted a protocol described by Ingersoll et al. (30) to further identify these subpopulations.

**Figure 4:**
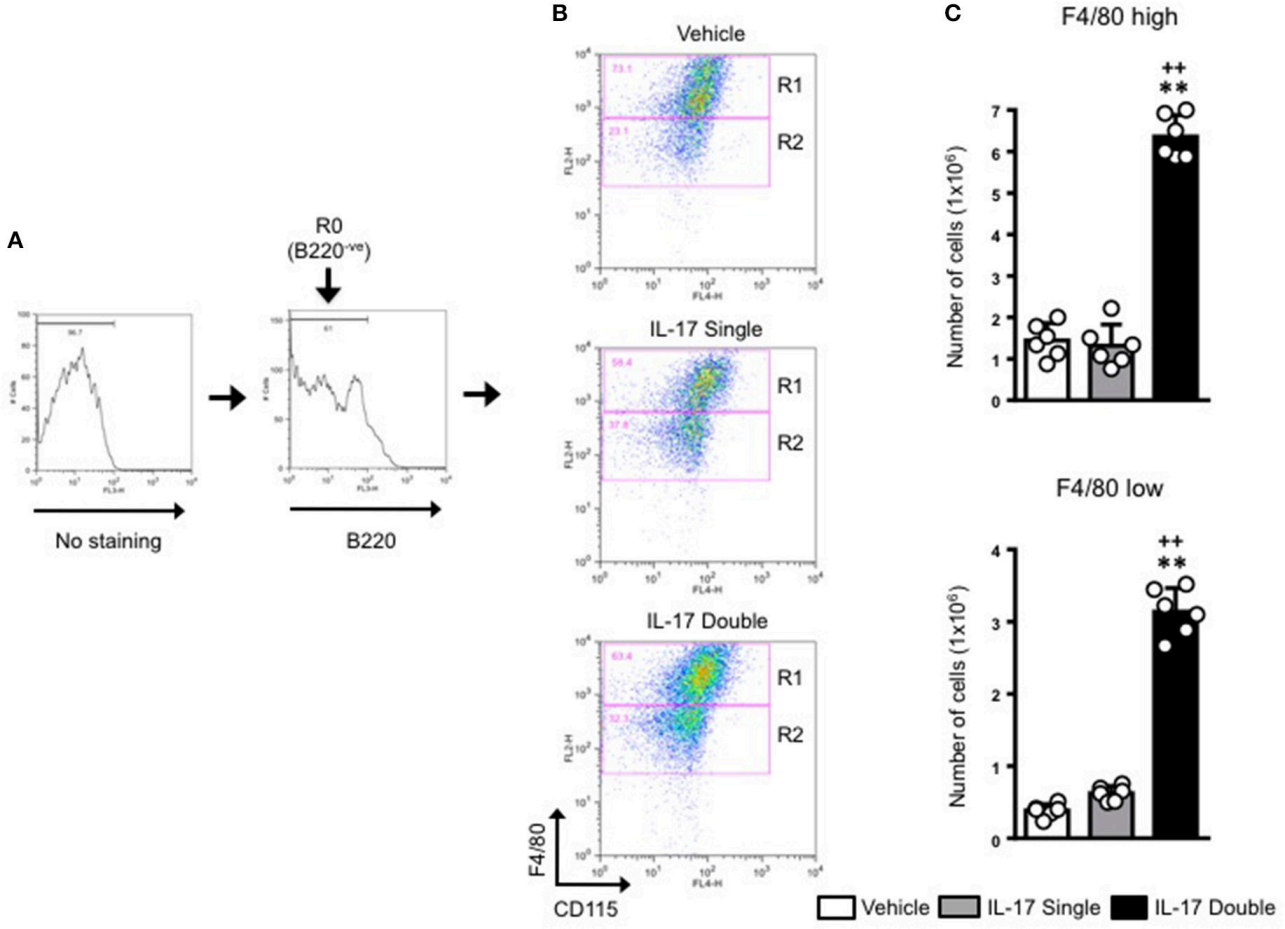
Quantification and gating strategy applied to identify monocyte populations in mice receiving single or double injection of IL-17 in the air pouch. **(A)** Cells obtained from vehicle, IL-17 single or double-injected air pouches were washed, stained as detailed in section Materials and Methods and analyzed by FACS. After first establishing the gate for background fluorescence (No staining), cells that were negative for B220 (R0; B220^−ve^) were gated and the plotted for F4/80 and CD115. **(B)** Gated cells were next divided in F4/80^high^-CD115^+ve^ (R1) and F4/80^low^-CD115^+ve^ (R2) positive cells as shown in the figure. Dot plots are from a single mouse and are representative of three separate experiments with *n* = 7 mice. **(C)** Bar graphs show the total number of F4/80^high^-CD115^+ve^ (gate R1) and F4/80^low^-CD115^+ve^ (gate R2) positive cells obtained from vehicle, IL-17 single or double-injected mice. Data are mean ± SEM, *n* = 6 animals per group of a single experiment and are representative of three independent experiments with similar results. Statistical analysis was conducted by one-way ANOVA with Bonferroni's multiple comparisons correction, **p < 0.01 vs. vehicle injection; ^++^*p* < 0.01 vs. IL-17 single injection.

For all samples, we first gated on the B220^−ve^ population and then plotted for F4/80 and CD115 expression (Figure 4A). This staining identified two populations: F4/80^high^/CD115^+ve^ (gated in R1) and F4/80^low^/CD115^+ve^ (gated in R2) (Figure 4B). As the expression level of F4/80 is commonly correlated with the degree of maturation of monocyte/macrophages (31, 32), we labeled the R1 population as macrophages and the R2 population as monocytes. Our results show that in double-IL-17-injected mice, the majority of the cells recovered were CD115^+^ and F4/80^+^ with other cells with intermediate or lower expression of both markers. These values were strengthened by an 0.89 % of F4/80^+high^/CD115^+ve^ found in the staining for the isotype control antibody (Supplementary Figure 5). Converting cell percentages in gated population in absolute cell number (Figure 4B), it was evident that in mice which received a double injection of IL-17, both R1 and R2 gated populations were significantly (*p* < 0.01) higher compared to mice that had received a single injection of IL-17 or vehicle (Figure 4C).

**Figure 5:**
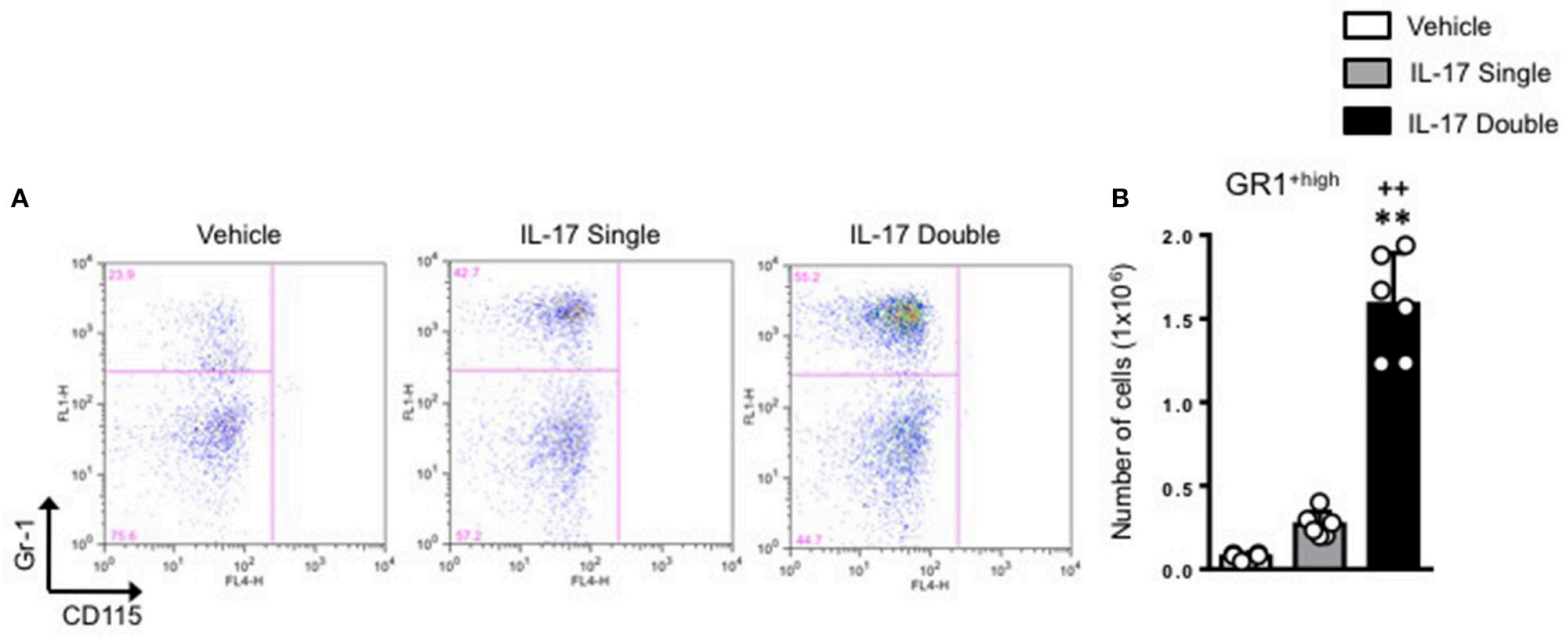
Elevated pro-inflammatory monocyte recruitment in mice receiving double injection of IL-17. **(A)** F4/80^low^-CD115^+ve^ positive cells (gate R2 described in Figure 4) obtained from vehicle, IL-17 single or double-injected mice were plotted for Gr-1 and CD115. Dot plots are from a single mouse and are representative of three separate experiments with *n* = 7 mice. **(B)** Bar graph shows the total number of GR-1^high^-CD115^+ve^ positive cells obtained from vehicle, IL-17 single or double-injected mice. Data are mean ± SEM, *n* = 6 animals per group. Statistical analysis was conducted by one-way ANOVA with Bonferroni's multiple comparisons correction, **p < 0.01 vs. vehicle injection; ^++^*p* < 0.01 vs. IL-17 single injection.

We next plotted R2^+ve^ populations for GR-1 and CD115 as the level of expression of Ly6C antigen (recognized by the anti–Gr-1 clone RB6-8C5) would allow us to distinguish GR-1^+high^ inflammatory monocytes from GR-1^+low^ patrolling monocytes. As shown in Figure 5A, there was a clear enrichment (55.2%) of GR-1^+high^ inflammatory monocytes in mice receiving a double injection of IL-17 compared to vehicle (29.9%) or single (42.7%) IL-17-injected.

Conversion of percentages in absolute numbers further highlighted these differences and showed a genuine role for IL-17 as a cytokine that favors the recruitment of inflammatory monocytes at the site of injury (Figure 5B).

### Double injection of IL-17 into the air pouch increases the release of IL-16 and trem-1 in the inflammatory fluid

**Figure 6:**
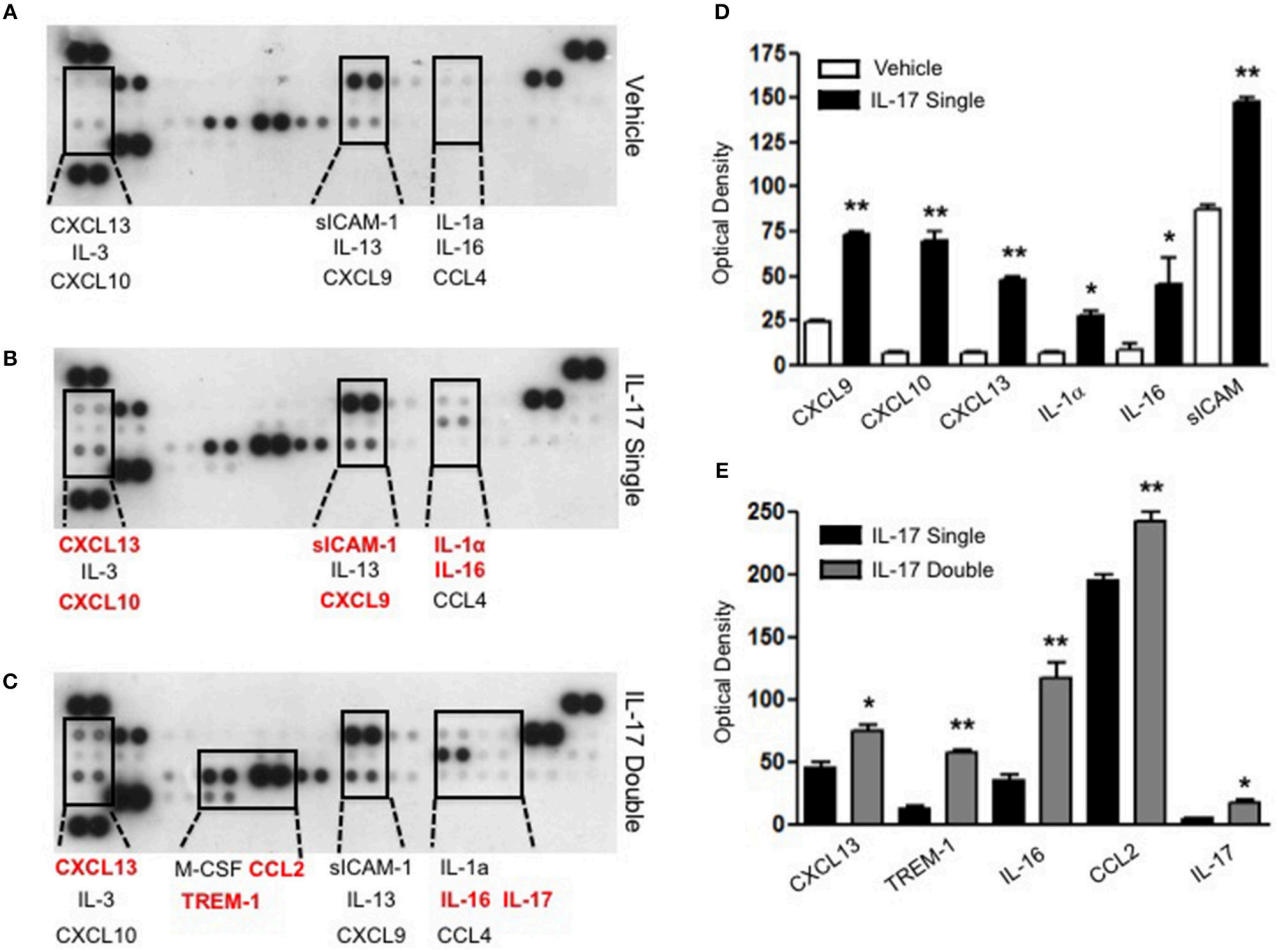
Survey of inflammatory mediators collected from mice receiving single and double injection of IL-17 in the air pouch. Inflammatory fluids obtained from air pouch were assayed as described in Materials and methods using a Proteome Profiler cytokine array (R&D system). The insets highlighted by the dotted lines show a magnification of specific areas where a signal was detected. The factors shown in red are mediators that are higher in the fluids obtained from **(B)** single and **(C)** double IL-17-injected mice compared to **(A)** vehicle control and single IL-17-injected mice, respectively. **(D–E)** Lavage fluid from a total of 7 mice per condition was pooled for each condition and run on a single blot. The bar graph shows the densitometric analysis of the arrays showed in **(A–C)**. Bars show mean changes ± S.E.M of the densitometric values obtained as a delta of increase in the O.D./mm^2^ compared to background. Statistical analysis was conducted by Students *T*-Test, *p < 0.05 and **p < 0.01 vs. **(D)** vehicle injection and **(E)** single IL-17-injected mice.

**Figure 7:**
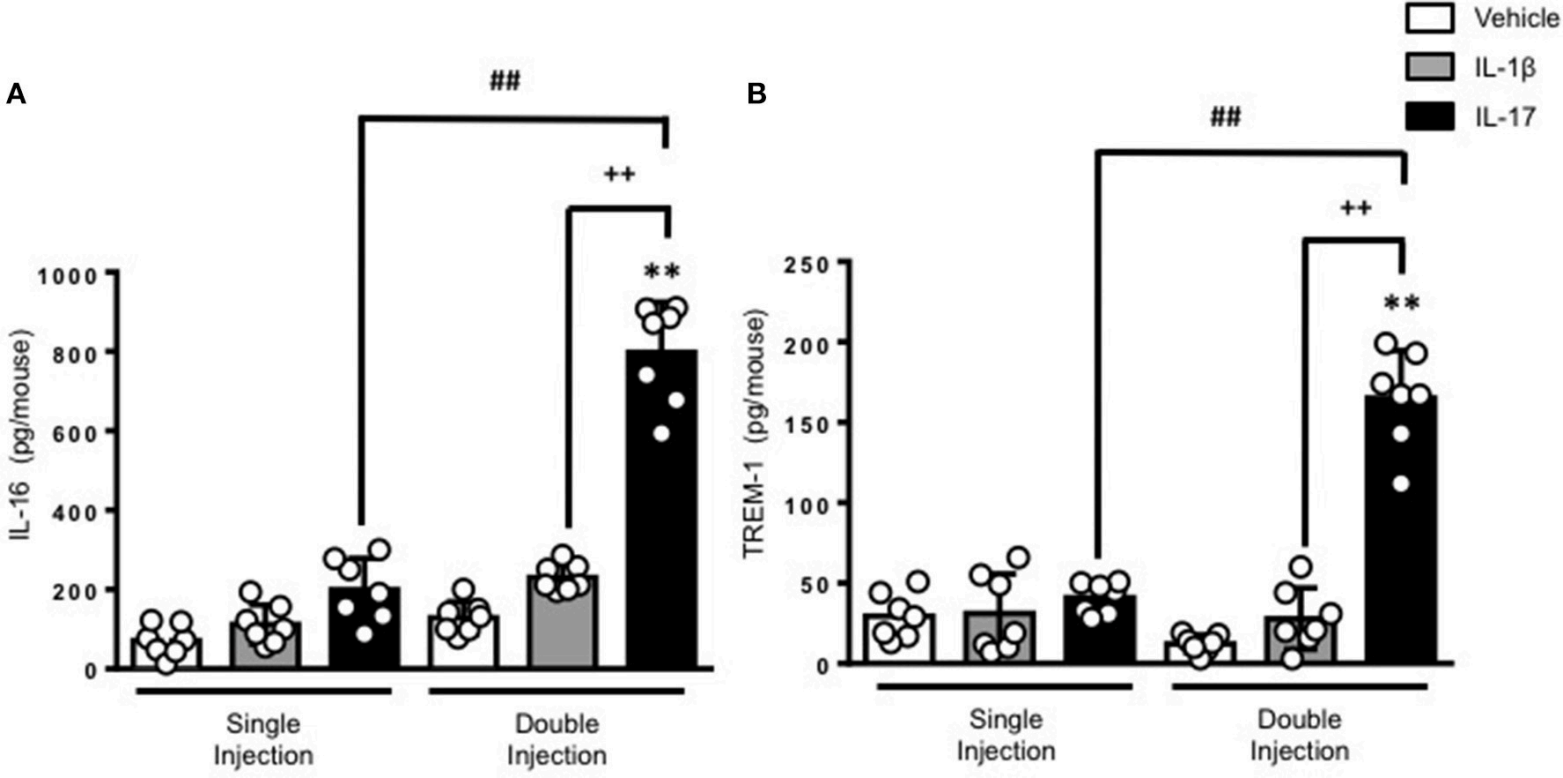
Levels of **(A)** IL-16 and **(B)** TREM-1 present in the inflammatory fluids of mice receiving single or double injection of IL-1β or IL-17 into the air pouch. Male C57/Bl6 mice received single or double injection of IL-1β or IL-17 (1.0 μg in 0.5 ml of 0.5% CMC) as described in Materials and Methods. The inflammatory fluids collected from the air pouch at 48 h were used to measure the levels of TNF-α and IL-6. Data are mean ± SEM, n = 7 animals per group of a single experiment and are representative of three independent experiments with similar results. Statistical analysis was conducted by two-way ANOVA with Bonferroni's multiple comparisons correction, **p < 0.01 vs. vehicle injection; ^*##*^*p* < 0.01 vs. IL-17 single injection; ^++^*p* < 0.01 vs. IL-1β double injection.

**Figure 8:**
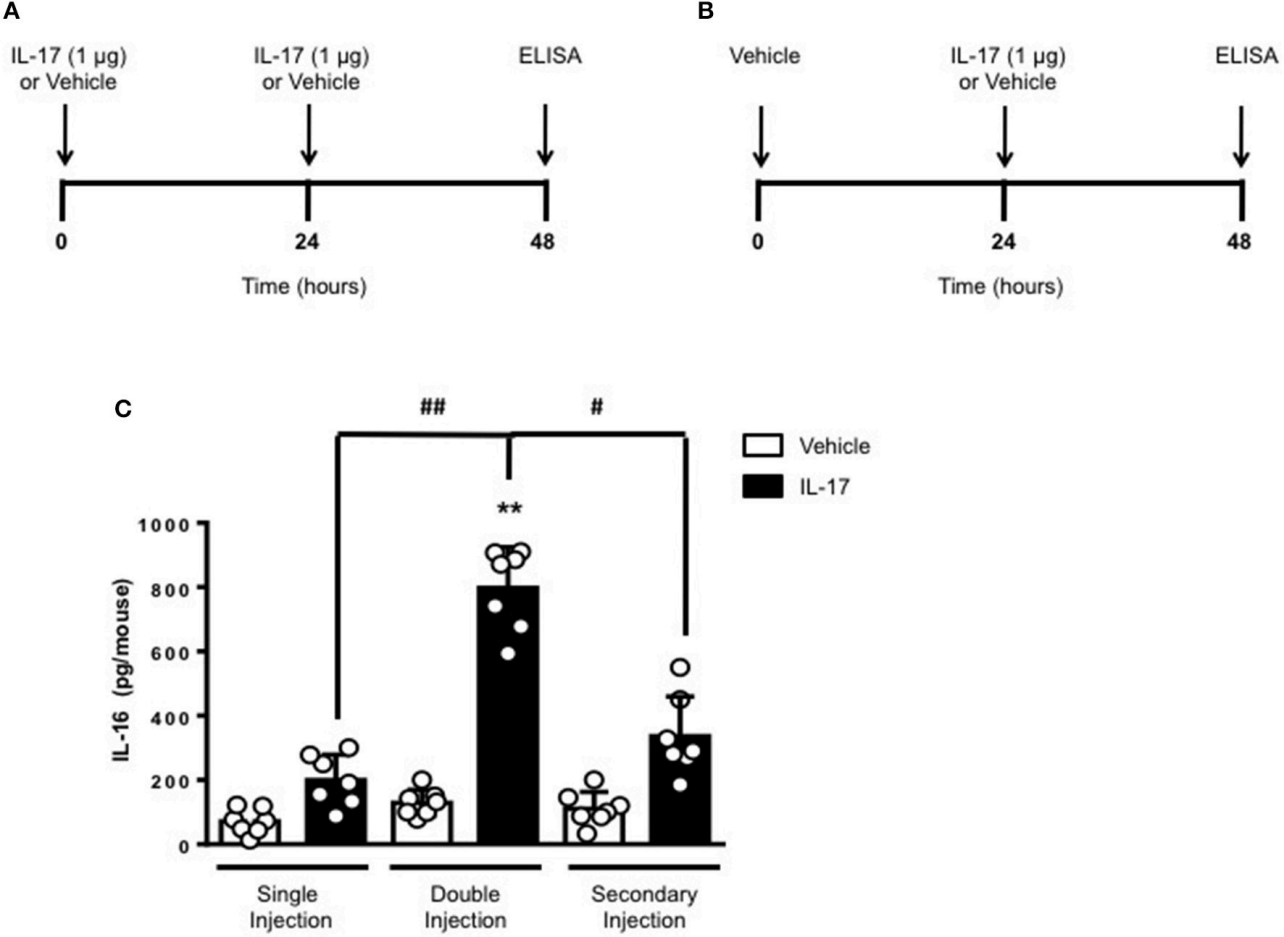
Levels of IL-16 in the inflammatory fluids of mice receiving IL-17 at different time-point. **(A)** Schematic representation of the experimental design used in this study. Male C57/Bl6 mice received one or two injections in the dorsal air pouch within 48 h. In the double injection protocol shown on the left **(A)**, mice received a first injection of IL-17 (1.0 μg in 0.5 ml of 0.5% CMC) at time 0 and a second injection of the cytokine after 24 h. In the secondary injection protocol (secondary injection) shown on the right (**B**), mice received an injection of IL-17 (1.0 μg in 0.5 ml of 0.5% CMC) 24 h post-vehicle (0.5 ml of 0.5% CMC) injection at time 0. In both protocols mice were culled at 48 h after model induction and then analyzed for the level of IL-16 into the pouch **(C)**. Data are mean ± SEM, *n* = 7 animals per group of a single experiment. Statistical analysis was conducted by one-way ANOVA with Bonferroni's multiple comparisons correction, **p < 0.01 vs. vehicle injection; ^#^*p* < 0.05 and ^##^*p* < 0.01 vs. IL-17 single or secondary injection.

**Figure 9:**
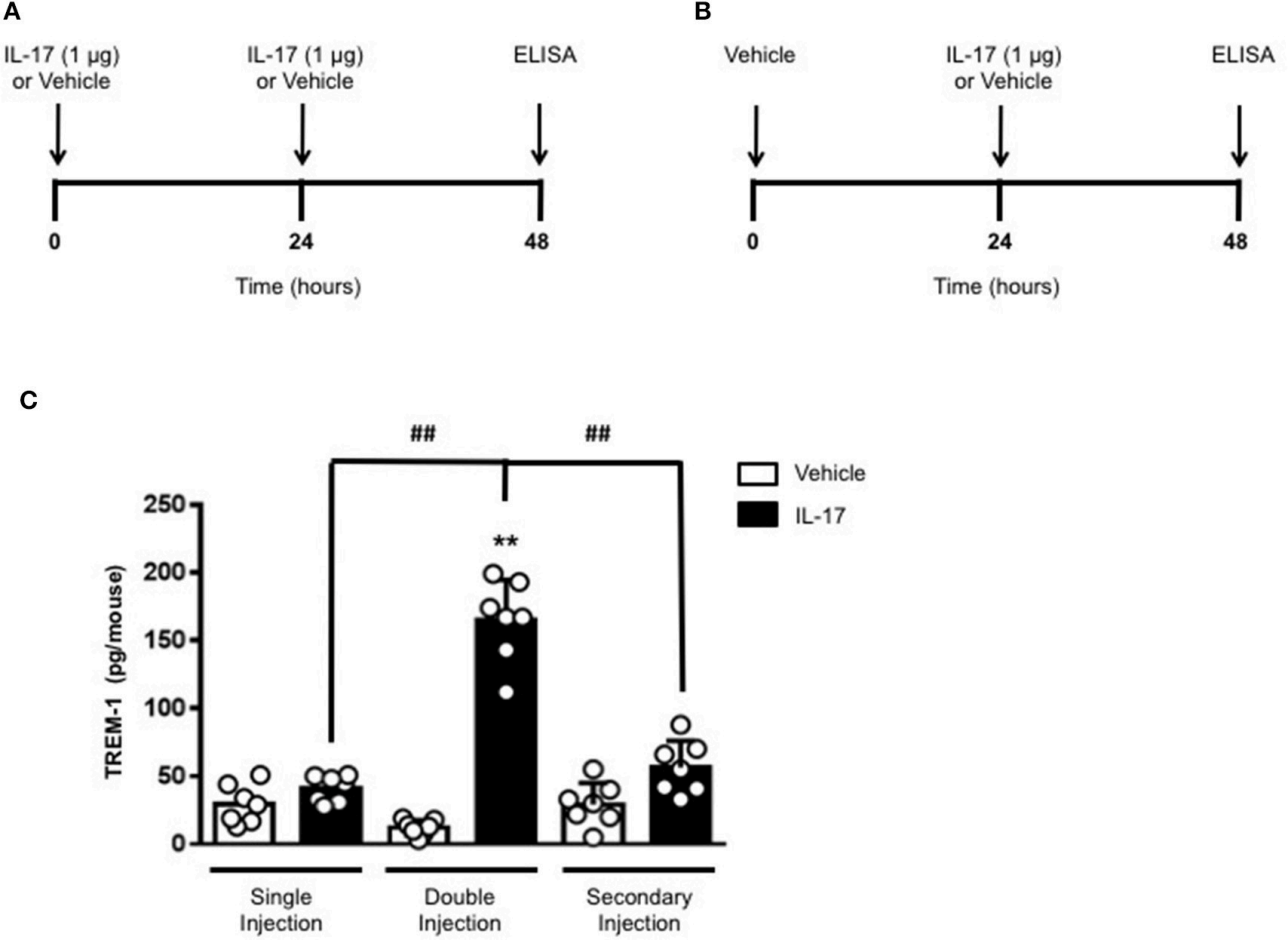
Levels of TREM-1 in the inflammatory fluids of mice receiving IL-17 at different time-point. **(A)** Schematic representation of the experimental design used in this study. Male C57/Bl6 mice received one or two injections in the dorsal air pouch within 48 h. In the double injection protocol shown on the left **(A)**, mice received a first injection of IL-17 (1.0 μg in 0.5 ml of 0.5% CMC) at time 0 and a second injection of the cytokine after 24 h. In the secondary injection protocol (secondary injection) shown on the right **(B)**, mice received an injection of IL-17 (1.0 μg in 0.5 ml of 0.5% CMC) 24 h post-vehicle (0.5 ml of 0.5% CMC) injection at time 0. In both protocols mice were culled at 48 h after model induction and then analyzed for the level of TREM-1 into the pouch **(C)**. Data are mean ± SEM, *n* = 7 animals per group of a single experiment. Statistical analysis was conducted by one-way ANOVA with Bonferroni's multiple comparisons correction, **p < 0.01 vs. vehicle injection; ^##^*p* < 0.01 vs. IL-17 single or secondary injection.

To gain some insights into other possible differences in the inflammatory response caused by a single or double injection of IL-17 we used an unbiased approach based on profiling cytokines and chemokines present in the inflammatory fluids using a pre-made protein array. As shown in Figure 6, the fluid obtained from a single administration of IL-17 showed the same profile of chemokines and cytokines present in vehicle (compare right and left panels, respectively). These included CXCL9, CXCL10, and CXCL13, soluble receptors such as soluble ICAM (sICAM) and TREM-1 and cytokines such as IL-16. However, densitometric analysis of the signals obtained from the two arrays (Figures 6A,B,D) showed that IL-17 single injection fluid had a specific increase in the following factors CXCL9, 10, and 13, IL-1 and IL-16 and sICAM compared to vehicle control. When comparing fluids from the single with the double we observed a selective increase (~4 time more) (Figures 6C,E) in CCL2, IL-16, and TREM-1. To further confirm the validity of these semi-quantitative findings and their specificity, we performed a standard ELISA for IL-16 and TREM-1 using the fluids obtained from multiple experiments including those with a single and double injection of IL-1β. As shown in Figure 7, only the fluids obtained from mice receiving a double injection of IL-17 showed a substantial increase of both cytokines with IL-16 being 5 times and TREM-1 about 6 times higher than vehicle. Consistent with the results obtained in Figure 6, there was also a doubling of the levels of IL-16 in the fluid of mice injected with a single dose of IL-17 mice compared to vehicle. However, these differences did not reach statistical significance. To ensure that the effects observed were a result of repeated exposure, and not the same as the acute response, we included mice injected with vehicle at time 0 and then IL-17 or Vehicle (secondary injection) at 24 h prior to culling and analyzing of both cytokines at 48 h (Figures 8, 9). We found that there was not a significant increase in terms of IL-16 and TREM-1 compare to vehicle, however, their level was still significantly decreased in mice which had a received double injection of IL-17 when compared.

## Discussion

The last decade has given birth to a “firework” of cytokines that have all been reported to play a significant role in inflammation (33–35). Some of these cytokines have been proposed to be cell signature e.g., to be specifically produced by a unique type of cell to the point that the cells were named after the cytokine (36, 37). Yet, these initial findings have been soon confuted as neither the cytokine nor the cells were that “unique” after all. This certainly is the case of IL-17 which was initially proposed to be specifically produced by autoimmune pro-inflammatory Th17 cells and later discovered to be produced at much higher level by non-Th17 cells (38–40)—to play host-protective functions against fungal infection (41)—and to be co-produced with anti-inflammatory cytokines such as IL-10 (42).

We have been interested in IL-17 from another perspective and we argued that studying the biological effects of this cytokine *per se*—rather than the producing cells—would help us gain more insight in its role in chronic inflammation (4–6, 43). In our previous studies (6) we have shown that recombinant IL-17 was unable to initiate an inflammatory response when injected in a tissue like the soft pad of the paw or into the peritoneal cavity of mice. However, injection of the same cytokine in the pre-inflamed cavity of an air pouch provided a completely different picture e.g., a time-defined recruitment of neutrophils and inflammatory monocytes that faded away over 48 h (6). This was consistent with existing literature proposing IL-17 as a genuine amplifier of the immune response because of the recruitment at the site of chronic inflammation of classical early phase inflammatory cells like neutrophils (2, 3, 9).

This sustained recruitment of neutrophils—at the later stages of an inflammatory response—has been proposed to be IL-17's main contribution to chronic inflammation (44–48). Here we expanded these initial observations adding another dimension to our established experimental system: time. In other words, here we have investigated what would be the effect of a persistent “presence” of IL-17 in a pre-inflamed tissue and, to do so, we have administered recombinant IL-17 into the pouch twice at time 0 and 24 h after the single injection. Consistent with our proposed role of an “amplifier” cytokine, double administration of IL-17 results in higher infiltration of leukocytes compared to a single dose. We wanted to make sure that this was not just the results of “double dose” of the inflammatory agent injected in the pouch and we thus tested the effect of a double injection of IL-1β but the results did not show any significant increase in the recruitment of inflammatory cells.

We speculated if the double number of inflammatory leukocytes we found was just the result of a continual accumulation of neutrophils or if other cells were being recruited by the double injection of IL-17. Analysis of the CD115^+^/F4/80^+^ cells to identify monocyte/macrophage (32) provided us with two populations: CD115^+^/F4/80^high^ mature macrophage and CD115^+^/F4/80^low^ monocytes. Plotting the CD115^+^/F4/80^low^ against GR-1^+^ showed a well-defined population of CD115^+^/F4/80^low^/GR-1^high^ clearly distinguishable from the CD115^+^/F4/80^low^/GR-1^low^. Both populations were more abundant in IL-17-double injected mice compared to IL-17 single or vehicle double injected animals that appeared to be indistinguishable (see Figure 5).

GR-1 recognizes both Ly6C and Ly6G antigens (29, 49); therefore, it seems reasonable to define the former monocytic lineage population as Ly6C^high^ inflammatory monocytes and the latter as Ly6C^low^ patrolling monocytes (50). The exact functions of these two distinct cell populations requires further investigation but one possible explanation may be that the CD115^+^ GR-1^+^F4/80^low^ monocytes are recruited to the air pouch to contribute to inflammation development, whereas the GR-1^−^F4/80^high^ cells derive from patrolling Ly6C^low^ monocytes which differentiate into macrophages in the pouch to promote inflammation resolution presumably via apoptotic neutrophil efferocytosis and wound healing facilitation (51–53).

Studies in the literature, have reported that Ly6C^hi^ monocytes express higher levels of IL-17RA than Ly6C^lo^ monocytes which makes them a preferential cellular responder to the inflammatory effects of IL-17 (54). Consistent with this, studies have shown that IL-17A^−/−^ mice present a defective neutrophil *and* monocyte recruitment in model of urinary tract infection by uropathogenic *Escherichia Coli* (55). Studies on the same mice have also shown a delayed healing by Ly6C^lo^MHC-II^hi^ monocytes in models of wound healing (56) further providing a link between IL-17 and monocyte/macrophages.

We wanted to understand if there were other ways in which the inflammatory response caused by the double injection of IL-17 would differ from that of the single injection and whether, like in the case of the monocytes, there were other soluble inflammatory mediators that would specifically increase in this setting. Both classical inflammatory cytokine TNF-α and IL-6 almost doubled in their levels in IL-17 double injected but not IL-1β or vehicle double injected mice (Figure 2) but these were not the only inflammatory mediators that were markedly increased in the inflammatory fluids.

A number of CXC and CC chemokines as well as sICAM-1 were induced upon single administration of IL-17 in comparison to the vehicle-treated animals (Figure 6). This is in line with earlier published work where IL-17 was shown to upregulate the secretion of pro-inflammatory chemokines such as CXCL2, CXCL10, CCL20, and CCL5 (55). Surprisingly, we did not detect any IL-8 in the air pouch which would justify the neutrophil recruitment in our model but instead detected the monocyte-derived chemokine CXCL10 and CCL4 and the lymphocyte recruiting CXCL9 and CXCL13. Interestingly, when we compared the single with the double injection of IL-17 we observed a 4-fold increase in the levels of two specific inflammatory mediators: IL-16 and TREM-1 (Figures 7, 8).

TREM-1 is a DAP12-associated receptor expressed on neutrophils and monocytes (57) that plays a key role in the protecting the host against fungal allergens (58), parasites (59) as well as in bacterial sepsis (60, 61), collagen-induced arthritis (62), and inflammation-associated tumor development (63). Interesting studies have also suggested that TREM-1 is involved in neutrophil migration across the epithelium (64) and macrophage infiltration at pathological sites (63) thus providing a possible explanation for the amplified inflammatory response we observed in our system.

IL-16 is classically considered as a CD4^+^ T cell chemoattractant cytokine whose role in inflammation has been demonstrated in a handful of inflammatory diseases such as experimental autoimmune encephalomyelitis (65, 66), rheumatoid arthritis (67), and allergy (68). A very interesting study by Cho et al. demonstrated that fibroblast-like synoviocytes from rheumatoid arthritis patients express higher levels of IL-16 compared to those from osteoarthritis patients (69). Most relevant to this study, IL-17 but not with IL-15, IL-1β, and IFN-γ were able to further increase the level of expression of IL-16 in fibroblast-like synoviocytes. The air-pouch is historically considered a model of “pannus formation” (70, 71) in the joint of rheumatoid arthritis patients. It would therefore be very tempting speculate that the lining tissue of the pouch—together with the infiltrated immune cells—is the biological source of IL-16.

What we think is most interesting and novel in this study is the possibility of the existence of two classes of inflammatory mediators: the inducers and the amplifiers. Such concept has already been proposed in the context of NF-κB signaling where IL-6 and TNF-α have been baptized as early inflammatory mediators as opposed to IFN-β related genes, cyclo-oxygenase (COX)-2 and inducible nitric oxide synthase (iNOS) (72, 73) being the late contributors to the sustaining of the inflammatory response. The consolidation of this hypothesis and classification would be important from translational point of view as drugs targeting the amplifiers might provide a better solution for several chronic inflammatory diseases as it would keep the response of the host to infection intact. This indeed is one of the main limitations of anti-TNF-α therapy that while effective in a wide variety of autoimmune disorders, it carries the risk of an impaired effective response to serious infections including tuberculosis (74).

## Author contributions

FM, AI, FR, ML, and MB performed the experiments. FDA designed the study, drafted, wrote the manuscript. AI, FDA, and FM edited and revised the manuscript.

### Conflict of interest statement

The authors declare that the research was conducted in the absence of any commercial or financial relationships that could be construed as a potential conflict of interest.

## Supplementary material

Supplementary Figure 1 Differential leukocyte counts after double injection of IL-17. **(A)** Schematic representation of the experimental design used in this study. Male C57/Bl6 mice received two injections of IL-17 (1.0 μg in 0.5 ml of 0.5% CMC) at time 0 and 24 in the dorsal air pouchs. **(B)** Differential cell count of inflammatory cells recovered from the air pouch of mice that have received double injection of IL-17. Data are mean ± SEM, *n* = 7 animals per group of a single experiment. Statistical analysis was conducted by Students *T*-Test, ^**^*p* < 0.01 when compared to vehicle.

Supplementary Figure 2 Effects of secondary and double injection of IL-17 in the air pouch. **(A)** Schematic representation of the experimental design used in this study. Male C57/Bl6 mice received one or two injections in the dorsal air pouch within 48 h. In the double injection protocol shown on the left **(A)**, mice received a first injection of IL-17 (1.0 μg in 0.5 ml of 0.5% CMC) at time 0 and a second injection of the cytokine after 24 h. In the secondary injection protocol (secondary injection) shown on the right **(B)**, mice received an injection of IL-1β or IL-17 (1.0 μg in 0.5 ml of 0.5% CMC) 24 h post-vehicle (0.5 ml of 0.5% CMC) injection at time 0. In both protocols mice were culled at 48 h after model induction and then analyzed for the number of inflammatory cells migrated into the pouch. **(C)** Number of inflammatory cells recovered from the air pouch of mice that have received a single or double injections of CMC vehicle (Vehicle; 0.5 ml) or the indicated inflamogens (1.0 μg in 0.5 ml of 0.5% CMC). Data are mean ± SEM, *n* = 7 animals per group of a single experiment. Statistical analysis was conducted by one-way ANOVA with Bonferroni's multiple comparisons correction, ^**^*p* < 0.01 vs. vehicle injection; ^#^*p* < 0.05 vs. IL-17 secondary injection; ^++^*p* < 0.01 vs. IL-1β secondary injection.

Supplementary Figure 3 Effects of single and double injection of IL-17 in the air pouch at different time-point. Male C57/Bl6 mice received different IL-17 injections in the dorsal air pouch within 48 h. In the double injection protocol, mice received a first injection of IL-17 (1.0 μg in 0.5 ml of 0.5% CMC) at time 0 and a second injection of the same inflamogen at the same concentration after 24 h **(A)**. In the single injection protocol, mice received a single IL-17 (2.0 μg in 0.5 ml of 0.5% CMC) at time 0 **(B)**. In both protocols mice were culled at 48 h after the first injection and then analyzed for the number of inflammatory cells migrated into the pouch. **(C)** Number of inflammatory cells recovered from the air pouch of mice that have received a single or double injections of CMC vehicle (Vehicle; 0.5 ml) or the indicated inflamogens (1.0 and 2.0 μg in 0.5 ml of 0.5% CMC). Data are mean ± SEM, *n* = 7 animals per group of a single experiment. Statistical analysis was conducted by two-way ANOVA with Bonferroni's multiple comparisons correction, ^**^*p* < 0.01 vs. vehicle injection; ^##^*p* < 0.01 vs. IL-17 single (2 μg) injection.

Supplementary Figure 4 Levels of IL-17 present in the inflammatory fluids of mice receiving a single injection of the cytokine into the air pouch. Male C57/Bl6 mice received a single injection of IL-17 (1.0 μg in 0.5 ml of 0.5% CMC) as described in section Materials and Methods **(A)**. The inflammatory fluids collected from the air pouch at 4, 24, and 48 h were used to measure the levels of IL-17. **(B)** Bars show mean values ± S.E.M of a single experiment with *n* = 7 independent mice for each time-point.

Supplementary Figure 5 Quantification and gating strategy applied to identify potential F4/80^high^-CD115^+ve^ and F4/80^low^-CD115^+ve^ positive cells with isotype control antibody. Cells obtained from air pouches were washed, stained as detailed in Materials and Methods and analyzed by FACS for detect F4/80^high^-CD115^+ve^ and F4/80^low^-CD115^+ve^ positive cells after the staining with isotype control antibody. Dot plots are from a single mouse and are representative of three separate experiments with *n* = 7 mice.
