# Supplementary figures and images for: Repetitive Exposure of IL-17 Into the Murine Air Pouch Favors the Recruitment of Inflammatory Monocytes and the Release of IL-16 and TREM-1 in the Inflammatory Fluids
Source: Front Immunol. 2018 Nov 30;9:2752. doi: 10.3389/fimmu.2018.02752 (PMC6284009; doi:10.3389/fimmu.2018.02752)

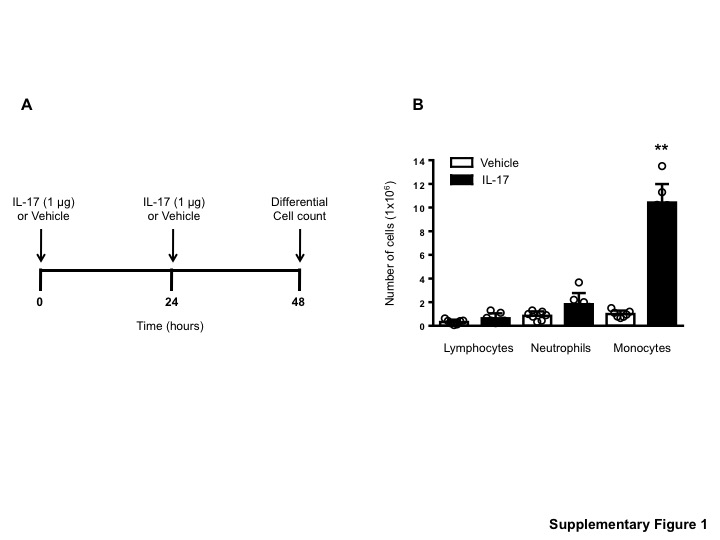

Supplement: Supplementary Figure 1 — Differential leukocyte counts after double injection of IL-17. (A) Schematic representation of the experimental design used in this study. Male C57/Bl6 mice received two injections of IL-17 (1.0 μg in 0.5 ml of 0.5% CMC) at time 0 and 24 in the dorsal air pouchs. (B) Differential cell count of inflammatory cells recovered from the air pouch of mice that have received double injection of IL-17. Data are mean ± SEM, n = 7 animals per group of a single experiment. Statistical analysis was conducted by Students T-Test, **p < 0.01 when compared to vehicle. [file Image_1.jpeg]

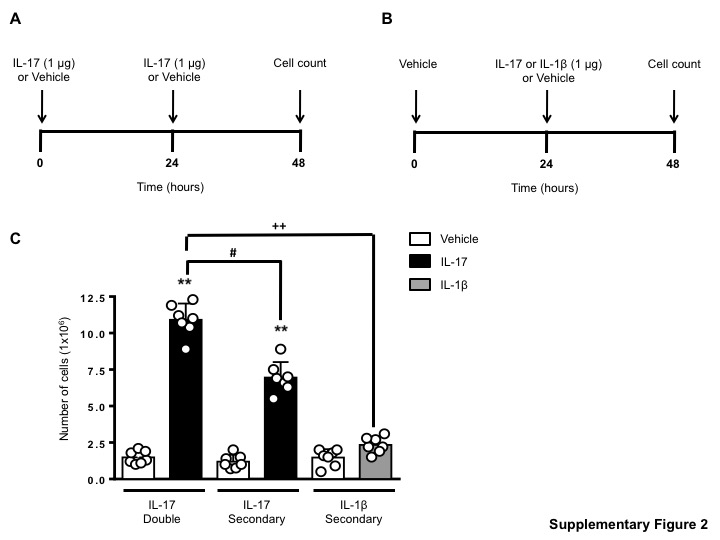

Supplement: Supplementary Figure 2 — Effects of secondary and double injection of IL-17 in the air pouch. (A) Schematic representation of the experimental design used in this study. Male C57/Bl6 mice received one or two injections in the dorsal air pouch within 48 h. In the double injection protocol shown on the left (A), mice received a first injection of IL-17 (1.0 μg in 0.5 ml of 0.5% CMC) at time 0 and a second injection of the cytokine after 24 h. In the secondary injection protocol (secondary injection) shown on the right (B), mice received an injection of IL-1β or IL-17 (1.0 μg in 0.5 ml of 0.5% CMC) 24 h post-vehicle (0.5 ml of 0.5% CMC) injection at time 0. In both protocols mice were culled at 48 h after model induction and then analyzed for the number of inflammatory cells migrated into the pouch. (C) Number of inflammatory cells recovered from the air pouch of mice that have received a single or double injections of CMC vehicle (Vehicle; 0.5 ml) or the indicated inflamogens (1.0 μg in 0.5 ml of 0.5% CMC). Data are mean ± SEM, n = 7 animals per group of a single experiment. Statistical analysis was conducted by one-way ANOVA with Bonferroni's multiple comparisons correction, **p < 0.01 vs. vehicle injection; #p < 0.05 vs. IL-17 secondary injection; ++p < 0.01 vs. IL-1β secondary injection. [file Image_2.jpeg]

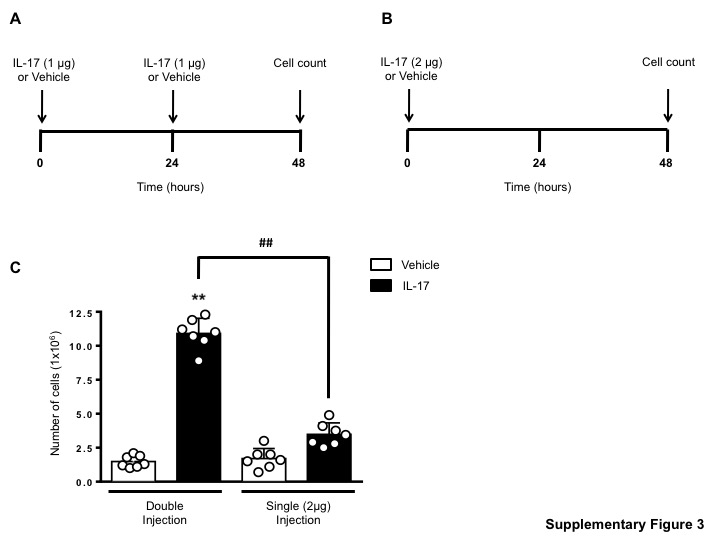

Supplement: Supplementary Figure 3 — Effects of single and double injection of IL-17 in the air pouch at different time-point. Male C57/Bl6 mice received different IL-17 injections in the dorsal air pouch within 48 h. In the double injection protocol, mice received a first injection of IL-17 (1.0 μg in 0.5 ml of 0.5% CMC) at time 0 and a second injection of the same inflamogen at the same concentration after 24 h (A). In the single injection protocol, mice received a single IL-17 (2.0 μg in 0.5 ml of 0.5% CMC) at time 0 (B). In both protocols mice were culled at 48 h after the first injection and then analyzed for the number of inflammatory cells migrated into the pouch. (C) Number of inflammatory cells recovered from the air pouch of mice that have received a single or double injections of CMC vehicle (Vehicle; 0.5 ml) or the indicated inflamogens (1.0 and 2.0 μg in 0.5 ml of 0.5% CMC). Data are mean ± SEM, n = 7 animals per group of a single experiment. Statistical analysis was conducted by two-way ANOVA with Bonferroni's multiple comparisons correction, **p < 0.01 vs. vehicle injection; ##p < 0.01 vs. IL-17 single (2 μg) injection. [file Image_3.jpeg]

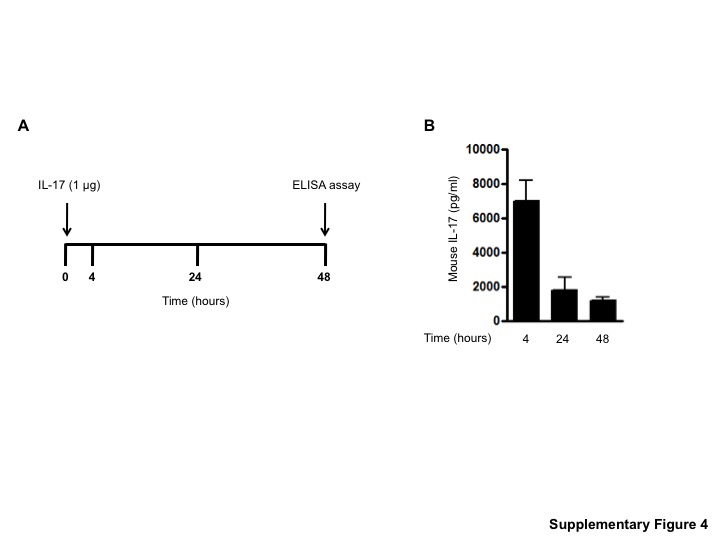

Supplement: Supplementary Figure 4 — Levels of IL-17 present in the inflammatory fluids of mice receiving a single injection of the cytokine into the air pouch. Male C57/Bl6 mice received a single injection of IL-17 (1.0 μg in 0.5 ml of 0.5% CMC) as described in section Materials and Methods (A). The inflammatory fluids collected from the air pouch at 4, 24, and 48 h were used to measure the levels of IL-17. (B) Bars show mean values ± S.E.M of a single experiment with n = 7 independent mice for each time-point. [file Image_4.jpeg]

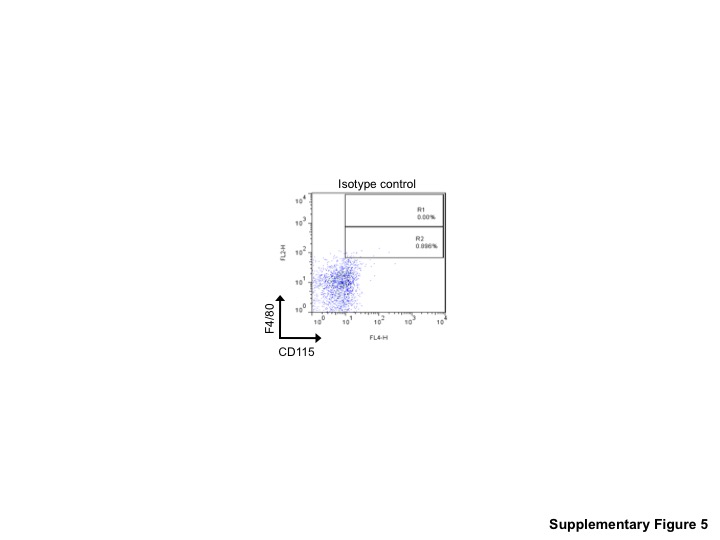

Supplement: Supplementary Figure 5 — Quantification and gating strategy applied to identify potential F4/80high-CD115+ve and F4/80low-CD115+ve positive cells with isotype control antibody. Cells obtained from air pouches were washed, stained as detailed in Materials and Methods and analyzed by FACS for detect F4/80high-CD115+ve and F4/80low-CD115+ve positive cells after the staining with isotype control antibody. Dot plots are from a single mouse and are representative of three separate experiments with n = 7 mice. [file Image_5.jpeg]
